# Adolescents’ quality of life in the light of mentalization and emotion regulation

**DOI:** 10.1192/j.eurpsy.2023.1502

**Published:** 2023-07-19

**Authors:** B. Tamás, B. Szabó

**Affiliations:** 1 ELTE Eötvös Loránd University PPK Institute of Psychology, ELTE Eötvös Loránd University; 2 ELTE Eötvös Loránd University PPK Institute of Psychology, ELTE Eötvös Loránd University Doctoral School of Psychology, Budapest, Hungary

## Abstract

**Introduction:**

According to research there is a negative association between emotion regulation, mentalization difficulties and quality of life among adolescents, but former research did not examine the relationship between these 3 constructs in a Hungarian adolescent sample.

**Objectives:**

The aim of our study was to examine the relationship between mentalization and emotion regulation with quality of life among 14- to 18-year-old adolescents.

**Methods:**

In our non-clinical, cross-sectional study 122 adolescents with informed consent answered a list of demographic questions, then completed the Reflective Function Questionnaire (RFQ-H), the Emotion Regulation Difficulties Questionnaire (DERS) and the Quality of Life Scale (ILK). In our mediator model we chose RFQ-H as the independent, DERS as the mediator and ILK as the dependent variable.

**Results:**

The first model was significant (*F*(1,120) = 28,79 *p* < 0,001, R^2^= 0,19), there was a significant relationship between mentalization disfunction and emotional regulation difficulties (*a*=0,39, *p*<0,01, β=0,44). The second model was significant as well (*F*(2,119= 30,48 *p* < 0,001, R^2^= 0,34), though the direct effect between mentalization difficulties and low quality of life was not significant (*c’*=0,02, *p*=0,73, β=0,03), the direct effect between emotion regulation difficulties and low quality of life was significant *(b*=0,58, *p*<0,01, β=0,57). The indirect effect between mentalization disfunction and low quality of life mediated by emotional regulation difficulties was also significant *ab* = 0,22 [0,13 – 0,33], β =0,25 [0,14 – 0,36]).

**Conclusions:**

Our results - taking the limitations into account - imply that emotional regulation mediates the relationship between mentalization and quality of life among the present-day, non-clinical, Hungarian adolescent sample, which could have practical implications.

**Disclosure of Interest:**

None Declared

